# Discussion on Prof. Keiller’s Paper

**Published:** 1892-03

**Authors:** 


DISCUSSION ON DR. KEILLER'S PAPER.

Dr. Wilkinson said he wished to thank Dr. Keiller for so interesting and instructive a paper. He appreciated and commended the instruments described, especially the small eyelet.

Prof. J. F. Y. Paine remarked that the strengthening of the vaginal portion was the most important improvement. One of the drawbacks of Barnes' bags, was this tendency of the bags to slip out, or entirely into the uterus. He would suggest a greater constriction of the cervical portion, to prevent this accident. He would also recommend that a larger size bag be made, for he sometimes found it necessary to use two of Barnes' bags at the same time. Dr. Paine thought the pocket of little use, for it was very difficult to introduce the bag with a sound. It was his practice to double the bag longitudionally, and introduce with dressing forceps. He did not use the stop-cock, but simply doubled the end of the tube to prevent collapse of the bag.

Dr. Fly stated that he had about discarded the use of Barnes' bag altogether. When rapid dilation was necessary, as in eclampsia, he used Goodell's dilator. In other cases for dilation, he introduces a cervical tampon of absorbent cotton, and packs the vagina tightly with the same material; for this procedure, the use of the speculum is necessary. Dr. Fly thought the modifications described by Dr. Keiller improvements over Barnes'.

Dr. Sampson said he had sometimes failed in the introduction of a Barnes bag, and now used metal dilators only, either Goodell's or Wylie's.

Dr. Hodges thought the advantages claimed for Dr. Keiller's modifications sustained; but it was his experience that rubber goods were soon rendered useless by this climate. He asked how Dr. Keiller preserved the specimens exhibited?

Prof. Keiller, in closing, thanked the members for the interest shown by their discussion. He thought the suggestion of Dr. Paine, to have an extra size bag, a good one. He did not think the modified bag would give any trouble by slipping into the uterus, on account of the thickened vaginal portion. He recommended that the bags be kept in tight tin boxes, to exclude light and air, in order to preserve them. If this were not successful, he thought the bags could be made of animal tissue, such as sheep's bladders. He did not think the elasticity of the material of much importance.

[NOTE.—The JOUUNAL is indebted to Dr. Vard H. Hulen, Secretary Galveston Medical Club, for the above.]

